# Antiviral Efficacy of Ribavirin and Favipiravir against Hantaan Virus

**DOI:** 10.3390/microorganisms9061306

**Published:** 2021-06-15

**Authors:** Jennifer Mayor, Olivier Engler, Sylvia Rothenberger

**Affiliations:** 1 Institute of Microbiology, University Hospital Center and University of Lausanne, CH-1011 Lausanne, Switzerland; jennifer.mayor@unil.ch; 2 Spiez Laboratory, Federal Office for Civil Protection, CH-3700 Spiez, Switzerland; Olivier.Engler@babs.admin.ch

**Keywords:** Hantaan virus, ribavirin, favipiravir, combination therapy

## Abstract

Ecological changes, population movements and increasing urbanization promote the expansion of hantaviruses, placing humans at high risk of virus transmission and consequent diseases. The currently limited therapeutic options make the development of antiviral strategies an urgent need. Ribavirin is the only antiviral used currently to treat hemorrhagic fever with renal syndrome (HFRS) caused by Hantaan virus (HTNV), even though severe side effects are associated with this drug. We therefore investigated the antiviral activity of favipiravir, a new antiviral agent against RNA viruses. Both ribavirin and favipiravir demonstrated similar potent antiviral activity on HTNV infection. When combined, the efficacy of ribavirin is enhanced through the addition of low dose favipiravir, highlighting the possibility to provide better treatment than is currently available.

## 1. Introduction

Orthohantaviruses (hereafter referred to as hantaviruses) are emerging negative-strand RNA viruses associated with two life-threatening diseases: hemorrhagic fever with renal syndrome (HFRS) and hantavirus cardiopulmonary syndrome (HCPS). Old World hantaviruses, including the prototypic Hantaan virus (HTNV) and Seoul virus (SEOV) are widespread in Asia where they can cause HFRS with up to 15% case-fatality. The New World hantaviruses Sin Nombre (SNV) and Andes (ANDV) are associated with HCPS in the Americas with up to 40% mortality. In Europe, Puumala virus (PUUV) causes nephropathia epidemica (NE), a milder form of HFRS, while Dobrava-Belgrade virus (DOBV) in the Balkans is associated with the more severe form of HFRS [1,2]. Rodents and insectivores represent the natural reservoir of hantaviruses, in which infections are persistent but asymptomatic. Humans’ infection is mainly transmitted by direct inhalation of contaminated rodent excreta [2,3], while human-to-human transmission has also been reported for ANDV via close contact with symptomatic persons [4,5,6,7].

Although a vaccine is available in several Asian countries (Hantavax^®^), the therapeutic options in Europe or the U.S.A. are limited to supportive care [8,9]. The only established antiviral drug with some efficacy in vitro and in vivo against hantaviruses is the nucleoside analogue ribavirin, approved in 1986, with broad-spectrum antiviral activity [10,11,12]. Ribavirin has some beneficial effects on HFRS patients, whereas treatment of HCPS with this drug remains inconclusive [1,13,14]. Moreover, the use of ribavirin is associated with side effects, including hemolytic anemia, and the drug cannot be applied in pregnant women. More effective antiviral therapies are therefore needed. A promising candidate that could be used in the future for hantavirus treatment is favipiravir, also known as T-705. It has been approved in 2014 in Japan to treat pandemic influenza infections and is reported to inhibit broad-spectrum RNA viruses, including ANDV and SNV [13,14,15,16,17,18]. Both ribavirin and favipiravir are prodrugs, which have to be metabolized for their conversion into efficient substrates for RNA incorporation, ribavirin-RTP and favipiravir-RTP [19,20,21,22]. Although both drugs are nucleoside analogues, they have been shown to have a distinct mode of action to inhibit influenza viral polymerase. Ribavirin causes GTP depletion, while favipiravir is used as an alternative nucleoside substrate by the viral polymerase, leading to the production of non-infectious viral particles [23].

In this study, we aimed to determine the antiviral efficacy of favipiravir compared to ribavirin and combined both drugs to boost their potency to protect from HTNV infection.

## 2. Materials and Methods

### 2.1. Cells and Viruses

Monkey kidney epithelial (Vero E6—Vero C1008, ATCC CLR 1586) cells were maintained in Biochrom minimum essential media (MEM) with Earle’s salts supplemented with 10% (*v*/*v*) FCS, 0.625% L-glutamine, 0.5% penicillin-streptomycin and 0.5% NEAA (Biochrom, Cambridge, UK) at 37 °C. For infection and viability studies, cells were seeded in 48-well plates at 50,000 cells/well and cultured for 16 h until cell monolayer formation. HTNV strain 76/118 [24] was propagated in Vero E6 cells for 15 days.

### 2.2. Inhibitor Treatment on Authentic Pathogenic Viruses, RNA Isolation, and RT-qPCR

Favipiravir (T-705) was from Toyama Chemical Co., Ltd. (Tokyo, Japan), diluted in DMSO at a concentration stock of 100 mM. For infection studies, favipiravir was tested at 5, 10, 15, 20 µM. Ribavirin was obtained from Sigma (Buchs, Switzerland), diluted at 10 mM in H_2_O and tested at 6.25, 12.5, 18.75, 25 µM. Drug interaction was tested in a 5 × 5 matrix in all possible combinations.

100% confluent cultures of Vero E6 cells were pre-treated with previously mentioned concentrations of favipiravir and/or ribavirin for 1 h. Cells were subsequently infected for 1 h with HTNV 76/118 strain (MOI of 1) in presence of the drug, washed once with medium, and incubated for 5 days with appropriate drug concentration. 100 µL of cell supernatants were collected and inactivated in 800 µL AVL-absolute EtOH solution. Viral RNA was isolated using MagNA Pure 96 Instrument (Roche, Mannheim, Germany). Quantification of viral RNA was performed using a real-time quantitative polymerase chain reaction (RT-qPCR) assay, specific for HTNV nucleocapsid coding region. TaqMan^®^ Fast virus 1-Step Master (4×), as well as HTNV PMGB1 probe (TCAATGGGGATACAACT) and HTNV primers (forward 5′-CATGGCATCHAAGACAGTKGG-3′, reverse 3′- TTWCCCCAGGCAACCAT-5′) were obtained from Applied Biosystems^®^ (Waltham, MA, USA). RT-qPCR was conducted in a LigthCycler^®^ 96 (Roche), following manufacturer’s instructions. For each experiment, one sample per condition was collected and experiments were conducted at least 3 times.

### 2.3. Therapeutic Index

To calculate the therapeutic index (TI), 100% confluent monolayers of Vero E6 cells were treated with ribavirin or favipiravir starting at 100 µM concentration, followed by a ~1.5-fold serial dilution for 5 days (concentrations tested: 0, 1, 2.5, 5, 10, 12.5, 25 µM). After 5 days, cells were treated with 1% Triton-100 (diluted in water) for 15 min at 37 °C in order to totally lyse the cells and obtain the background levels. The cytotoxicity of ribavirin and favipiravir was assessed using CellTiter-Glo^®^ Assay System (Promega, Madison, WI, USA), which determines the number of viable cells on the basis of ATP levels, with the TriStar LB 941 (Berthold Technologies, Bad Wildbad, Germany) luminometer. The median toxic dose (TD_50_) was calculated based on the cell viability values, while the median effective dose (ED_50_) on inhibition of HTNV replication by RT-qPCR. Therapeutic index was finally calculated as followed: TD_50_/ED_50_.

### 2.4. Data Analysis

The Bliss synergy analysis was performed using Synergy Finder software (SynergyFinder 2.0: visual analytics of multi-drug combination synergies, Helsinki, Finland [25]). Dose-response curves for ribavirin and favipiravir were generated and IC_90_ for each individual drug were calculated with GraphPad Prism 7 software (San Diego, CA, USA, www.graphpad.com) to perform the combination studies. The 5 × 5 matrix was designed around IC_90_ values and for each combination, percentage of inhibition was assigned in the dose-response matrix. In the synergy map, score and color are indicating the degree of either antagonism (green, decreased effect, score > −10), additivity (white, score from −10 to 10), or synergism (red, increased effect, score > 10).

### 2.5. Statistical Analysis

Graphical representation and statistical analysis were performed using GraphPad Prism 7 software. Data are means + SD (n = 3) and *p* values of <0.05 were considered statistically significant.

## 3. Results

### 3.1. Ribavirin and T-705 Demonstrate Similar Antiviral Activity against HTNV Infection

To assess antiviral drug activity of ribavirin and T-705 against HTNV infection, Vero E6 cells were treated with increasing concentration of both compounds prior to infection. Virus production in the cell supernatant was detected using a highly sensitive real-time quantitative polymerase chain reaction (RT-qPCR) 5 days post-infection.

**Figure 1:**
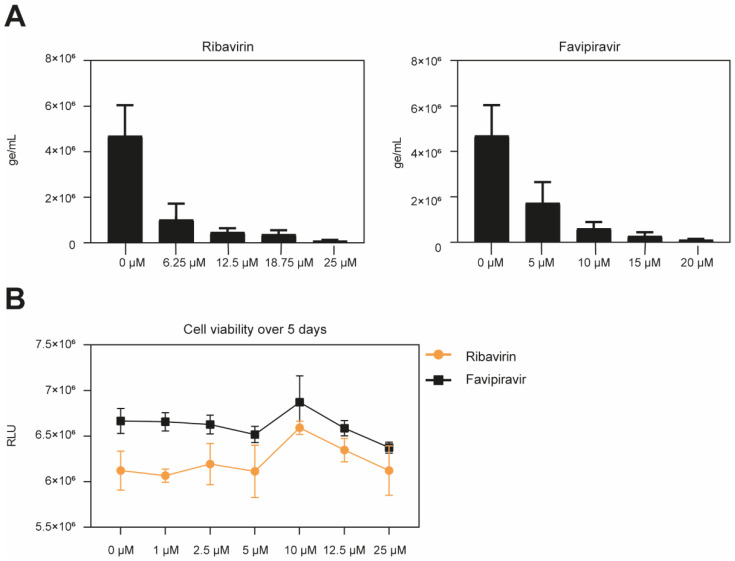
Antiviral activity of ribavirin and favipiravir. (**A**) Dose-response curve in Vero E6 cells treated with ribavirin, or favipiravir. Vero E6 cells were pre-incubated with ribavirin or favipiravir at the indicated concentrations for 1 h at 37 °C, followed by Hantaan virus (HTNV) infection for 1 h at 37 °C. Cells were subsequently washed once with medium and incubated with medium containing appropriate drug concentration for 5 days. At 5 dpi, the viral titer on the cell supernatant was determined by RT-qPCR. Data are means + SD (n = 3) of genome per mL. (**B**) Cell viability over 5 days. Monolayers of Vero E6 cells were treated with the indicated concentration of ribavirin and favipiravir under the assay conditions. After 5 days, intracellular ATP levels were measured using CellTiter-Glo^®^ assay. Data are means ± SD (n = 3) of Relative Light Units (RLU) of three independent experiments.

Ribavirin and favipiravir both inhibited HTNV infection in a dose-dependent manner. The IC_50_ of ribavirin corresponded to 2.65 μM and the IC_90_ of 10.49 μM, whereas favipiravir demonstrated an IC_50_ of 3.89 μM and IC_90_ of 9.79 μM (Figure 1A). The drugs applied under the assay conditions demonstrated no cytotoxic effect, as observed by a measurement of the cell viability (Figure 1B). Overall, both compounds inhibit HTNV infection with high potency.

### 3.2. Additive Effect between Ribavirin and T-705 in Inhibiting Hantavirus Replication

**Figure 2:**
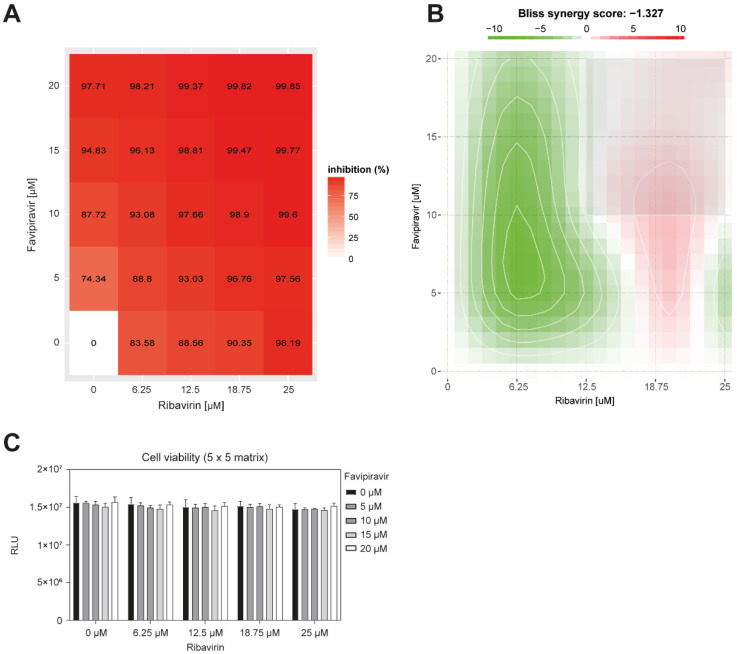
Additive effects of ribavirin with T-705. Vero E6 cells were pre-treated with a 5 × 5 compound combination matrix prior to infection with HTNV. At 5 dpi, the viral titer on the cell supernatant was determined by RT-qPCR. The compound combinations evaluated were ribavirin and T-705. (**A**) Dose-response matrix of favipiravir/ribavirin combinations. The values reported are expressed in percentage of virus production inhibition. Infection levels were assessed by RT-qPCR. Data are means of triplicate experiments. (**B**) Bliss synergy map. Color scale bar indicate mode and strength of interaction. Antagonism: less than −10; additive: from −10 to 10; synergism: larger than 10. Data are means of three independent experiments. (**C**) Cell viability over 5 days. Monolayers of Vero E6 cells were treated with the indicated concentration of ribavirin and/or favipiravir under the assay conditions and viability was measured by the Cell-Titer Glo^®^ assay. Data are means + SD (n = 3) of relative light units (RLU).

As ribavirin is the standard treatment for HFRS disease, and that T-705 is a potent novel antiviral against HTNV infection, we next investigated whether T-705 would result in a synergistic, additive, or antagonistic effect with ribavirin. We designed a 5 × 5 concentration matrix around the IC_90_ values of both drugs (Figure 2A). Vero E6 cells were treated for 60 min with increasing concentration of ribavirin and favipiravir, followed by 1 h infection with HTNV. Inoculum was then removed, and medium with appropriate drug concentration was added for 5 days. Virus production in the cell supernatant was determined by RT-qPCR.

To evaluate antagonistic or synergistic effects, we used the Bliss independence model, which states that two drugs have an independent effect, and their combination is calculated based on the probability of independent events [26,27,28]. As ribavirin and favipiravir have been demonstrated to block infection via distinct modes of action, they are considered to act independently [23]. All combinations demonstrated a strong antiviral efficacy (Figure 2B), and the drug combinations demonstrated no cell toxicity (Figure 2C). The highest antiviral activity showing a decrease of HTNV particles from 5.8 × 10^6^ to 6.3 × 10^4^ genome/mL and corresponding to >99% of inhibition, is observed with the drug combination 12 μM favipiravir/18.75 μM ribavirin. The Bliss synergy score of −1.327 argues for an additive effect.

We then calculated the toxicity index (TI) to determine the relative safety of the drugs ribavirin and favipiravir (Table 1). Vero E6 cells were treated under the assay conditions from 1 to 100 μM. 5 days post-treatment, cells were lysed with 1% Triton-100 to obtain the background values. Ribavirin is the compound demonstrating the highest relative safety with a therapeutic index of 4.9 × 10^5^:1, while favipiravir has a lower therapeutic index of 4.3 × 10^3^:1.

## 4. Discussion

Hantaan virus (HTNV) is responsible of HFRS, a severe human disease and the therapeutic approaches currently used are limited to supportive care, highlighting the need for novel therapeutic strategies against pathogenic HTNV.

The antiviral used currently, ribavirin, improves HFRS disease outcome when given early in infection, while its effectiveness to treat HCPS is less clear [29,30]. Moreover, ribavirin is associated with some side effects, including hemolytic anemia, and cannot be applied in pregnant women. Another more recently approved antiviral molecule, demonstrating broad-spectrum antiviral activity on RNA viruses, is favipiravir (T-705) [16]. This compound has been approved for influenza treatment in Japan since 2014 [31] and was administered during the West Africa Ebola outbreak as an emergency antiviral strategy [32,33]. Favipiravir was previously shown to have an inhibitory effect on RNA viruses, among them the arenaviruses Lassa (LASV) and Junín, as well as SNV and ANDV, in vitro and in vivo [13,18,34,35,36,37]. Favipiravir could thus represent a potent antiviral to treat HFRS. In this study, we first aimed to evaluate and compare the antiviral effect of favipiravir and ribavirin. Both drugs show high potency and similarly inhibit HTNV infection with an IC_50_ around 3 µM and an IC_90_ around 10 µM. However, as favipiravir was demonstrated to have higher antiviral activity in vivo for several viruses, including Crimean–Congo hemorrhagic fever, Lassa, and Ebola viruses [27,37,38], we may consider T-705 as a stand-alone treatment.

Regarding the biochemical mechanism, ribavirin and favipiravir have been shown to have distinct modes of action to inhibit influenza viral polymerase [23]. As both drugs block infection via different mechanisms, we tested the impact of the combination of the two drugs on HTNV infection. The drug concentrations used in these experiments were selected based on the IC_90_ values, as previously determined for LASV infection in animal models to observe an effective antiviral activity [26]. Our results indicate that ribavirin and favipiravir have an additive interaction when suboptimal doses were combined compared to treatment with each drug alone. These in vitro data suggest that favipiravir could decrease the ribavirin concentration needed in vivo, and consequently lower the associated side effects. One limitation in using ribavirin or favipiravir as monotherapy is the virulence and high mutation rates of human pathogenic RNA viruses [38,39], which can lead to the emergence of viral resistance and thus decrease antiviral activity, as illustrated by HIV [40]. Therefore, the combination of antiviral treatment is a promising strategy.

The precise mode of action of both drugs on HTNV infection are yet to be elucidated. In analogy to other RNA viruses, one may speculate that ribavirin lowers GTP levels, increasing the use of the nucleoside analogue T-705, which in turn is incorporated into RNA strands to induce viral mutagenesis. The mechanism of favipiravir on HTNV still requires further investigation. It has been previously shown that ribavirin induces mutation into the viral genome of HTNV, leading to errors in viral replication and thus the production of non-infectious viral particles [41]. Although RT-qPCR allows a precise quantification of viral RNA, it remains to be determined whether the newly synthesized virion are infectious. In a follow-up study, we plan to count the number of infectious particles using focus assays, and perform a deeper analysis using high throughput sequencing in order to determine the type and frequency of the mutations after drug treatment [41]. These studies may allow us to better understand the different mechanisms of action of ribavirin and favipiravir in the context of HTNV infection.

Although further experiments are required to investigate the toxicity and the in vivo antiviral potency, the lack of an appropriate animal model exhibiting clinical signs of the HFRS disease renders this difficult [42]. On the other hand, Syrian hamsters are useful animal models for HCPS and recapitulate the clinical human disease [43,44,45,46]. A previous in vivo study on LASV demonstrated the potential of combined drug treatment to significantly increase the survival rate in mice [26]. Most interestingly, two human patients infected with LASV were successfully treated with a combination of ribavirin/favipiravir [47]. These studies open a new avenue of research and indicate that a combination of ribavirin/favipiravir could constitute a potential treatment of the diseases caused by HTNV and others. The risk of resistance triggered by the use of a single replication inhibitor has to be considered. A promising line of approach is therefore the combination of antiviral drugs that target different steps of the viral lifecycle. Attacking the virus from different angles make it less likely for drug-resistance to occur. Several molecules inhibiting HTNV entry were identified in our laboratory, but their uses still need further investigation in combinatory studies with replication inhibitors [48,49,50].

In the context of sporadic outbreaks caused by hantaviruses, as well as imported cases of infected patients, the diagnosis and clinical management remains a challenge. In Switzerland, two imported cases of ANDV infection and three of PUUV infection were recently reported [51,52]. Regarding HCPS cases, the two patients were a 55-year-old man and his 54-year-old wife returning from South America. The husband first developed fever on the day of their return to Switzerland, while he deteriorated and required non-invasive ventilator support, ANDV infection had been confirmed by RT-qPCR 8 days later. At this time point, his wife demonstrated no clinical symptoms, and the serology and RT-qPCR tests were negative. However, 28 days after her husband’s first symptoms, she also presented to the hospital with pronounced weakness, myalgia, and shortness of breath. On the second day, she was diagnosed positive for ANDV infection and remained 5 months in the intensive care unit, followed by pulmonary intermediate care for 1 month and another complete year in neuro-pulmonary rehabilitation. In contrast, after influenza-like symptoms of the mother and fatal PUUV infection of the father (45 years of age), the patients’ daughter received a successful 5-day course of oral ribavirin. A prophylactic treatment therefore represents a helpful protection for the other family members. In vitro studies demonstrating antiviral efficacy therefore lay the basis for considering an experimental therapy.

Early diagnosis, allowing rapid therapeutic intervention, will significantly improve the outcome of hantavirus disease. Lowering the viral load early in infection will provide the patient’s immune system precious time to mount an antiviral response that will control and ultimately clear infection. We expect that treatment with ribavirin and favipiravir for post-exposure prophylaxis and during early infection may reduce mortality and provide better treatment than is currently available.

## Figures and Tables

**Table 1:** Therapeutic index. The therapeutic index (TI) is defined as toxic dose (TD_50_)/effective dose (EC_50_).

	Toxic Dose (TD_50_)	Effective Dose (ED_50_)	Therapeutic Index (TI)
Ribavirin	1,306,801 µM	2.647 μM	4.9 × 10^5^
Favipiravir (T-705)	16,796 µM	3.888 μM	4.3 × 10^3^

## Data Availability

All data are available under request.
